# Supplementary material for: HIVprotI: an integrated web based platform for prediction and design of HIV proteins inhibitors
Source: J Cheminform. 2018 Mar 9;10:12. doi: 10.1186/s13321-018-0266-y (PMC5845081; doi:10.1186/s13321-018-0266-y)
Supplement: Supplementary file 2 — Additional file 2. Source code of HIVProtI web server. [file 13321_2018_266_MOESM2_ESM.zip › HIVprotI_Source-code/hivprotI/weka-3-6-6/doc/weka/gui/beans/DataSource.html]

DataSource


---


|  |  |  |
| --- | --- | --- |
| **Overview** \| **Package** \| **Class** \| **Tree** \| **Deprecated** \| **Index** \| **Help** \| **Weka's home** | |  |
| **PREV CLASS**   **NEXT CLASS** | **FRAMES**    **NO FRAMES**     **All Classes** |  |
| SUMMARY: NESTED \| FIELD \| CONSTR \| METHOD | DETAIL: FIELD \| CONSTR \| METHOD |  |


---


## weka.gui.beans Interface DataSource

**All Known Implementing Classes:**: AbstractDataSource, ClassAssigner, Filter, InstanceStreamToBatchMaker, Loader, PredictionAppender

---

``` public interface DataSource ```

Interface to something that is capable of being a source for data -
either batch or incremental data

**Since:**
:   1.0

**Version:**
:   $Revision: 1.2 $

**Author:**
:   Mark Hall

---

| **Method Summary** | |
| --- | --- |
| `void` | `addDataSourceListener(DataSourceListener dsl)`             Add a data source listener |
| `void` | `addInstanceListener(InstanceListener dsl)`             Add an instance listener |
| `void` | `removeDataSourceListener(DataSourceListener dsl)`             Remove a data source listener |
| `void` | `removeInstanceListener(InstanceListener dsl)`             Remove an instance listener |

**Method Detail**

### addDataSourceListener

```
void addDataSourceListener(DataSourceListener dsl)
```

:   Add a data source listener

    :   **Parameters:**: `dsl` - a `DataSourceListener` value

---


### removeDataSourceListener

```
void removeDataSourceListener(DataSourceListener dsl)
```

:   Remove a data source listener

    :   **Parameters:**: `dsl` - a `DataSourceListener` value

---


### addInstanceListener

```
void addInstanceListener(InstanceListener dsl)
```

:   Add an instance listener

    :   **Parameters:**: `dsl` - an `InstanceListener` value

---


### removeInstanceListener

```
void removeInstanceListener(InstanceListener dsl)
```

:   Remove an instance listener

    :   **Parameters:**: `dsl` - an `InstanceListener` value


---


|  |  |  |
| --- | --- | --- |
| **Overview** \| **Package** \| **Class** \| **Tree** \| **Deprecated** \| **Index** \| **Help** \| **Weka's home** | |  |
| **PREV CLASS**   **NEXT CLASS** | **FRAMES**    **NO FRAMES**     **All Classes** |  |
| SUMMARY: NESTED \| FIELD \| CONSTR \| METHOD | DETAIL: FIELD \| CONSTR \| METHOD |  |


---
